# Maternal Nutritional Status and Intrauterine Programming: Epigenetic Mechanisms and Their Impact on Offspring Health

**DOI:** 10.3390/nu18142220

**Published:** 2026-07-08

**Authors:** Klaudia Pawlikowska, Anna Koryszewska-Bagińska, Małgorzata Konieczna, Gabriela Olędzka

**Affiliations:** 1 Students Scientific Club Agar, Department of Medical Biology, Faculty of Health Sciences, Medical University of Warsaw, 00-575 Warsaw, Poland; 2 Department of Medical Biology, Faculty of Health Sciences, Medical University of Warsaw, 00-575 Warsaw, Poland; malgorzata.konieczna@wum.edu.pl (M.K.); gabriela.oledzka@wum.edu.pl (G.O.)

**Keywords:** maternal nutrition, epigenetics, developmental programming, foetal development, offspring health

## Abstract

Maternal nutritional status before and during pregnancy is an important modifiable factor that may influence foetal development and long-term offspring health. Both undernutrition and excessive maternal body weight have been associated with adverse metabolic, cardiovascular, neurodevelopmental and immune-related outcomes in offspring. This narrative review summarises selected experimental, clinical and epidemiological evidence on maternal nutritional status, intrauterine programming and offspring health. Relevant literature was identified by searching the PubMed, Scopus and Web of Science databases covering publications from 2000 to 2026, with the majority of included papers published after 2015. The search terms included maternal nutrition, undernutrition, obesity, intrauterine programming, DOHaD, epigenetics, DNA methylation, microRNA and offspring health. The available evidence suggests that maternal undernutrition is associated with intrauterine growth restriction (IUGR) and an increased risk of cognitive and metabolic disorders. In contrast, maternal overweight or obesity is associated with macrosomia, as well as an increased risk of obesity, cardiovascular disease and metabolic dysfunction in later life. Both extremes of maternal nutritional status may also negatively affect the psychological health of the offspring. These associations suggest a potential role for epigenetic mechanisms; however, causal conclusions from observational studies in humans remain limited. Overall, the findings highlight the importance of optimising maternal nutritional and metabolic status before and during pregnancy as a key strategy for promoting favourable intrauterine programming and improving long-term offspring health. A better understanding of the underlying epigenetic mechanisms could facilitate the development of targeted nutritional and preventive interventions. As a narrative review, this analysis is limited by the associative nature of human observational studies and the inherent challenges in separating intrauterine effects from postnatal environmental confounders.

## 1. Introduction

In recent years, increasing attention has been paid to maternal lifestyle and nutrition during pregnancy. The quality of a mother’s diet, her physical activity, alcohol consumption, and exposure to harmful substances have been widely studied. However, maternal body composition both before and during pregnancy remains relatively underestimated. Although this factor is modifiable, it plays a crucial role in foetal development. Importantly, this applies during the gestation period and the preconception period. Evidence from experimental and observational studies suggests that maternal nutrition may influence not only directly exposed offspring but potentially also later generations; however, true transgenerational inheritance should be distinguished from intergenerational exposure and remains difficult to confirm in humans [1]. The timing of nutritional disturbances is as important as their magnitude. The periconceptional period, the rapid growth of the placenta, and organ-specific sensitive periods such as the nephrogenic phase up to 36 weeks and cerebellar development during the last trimester can all be affected differently depending on whether there is undernutrition or overnutrition. Thus, any attempt to ensure optimum nutrition in pregnant women should take into consideration these crucial periods of pregnancy [2]. The concept of intrauterine programming was already introduced in the early twentieth century. It evolved from studies linking low birth weight with an increased risk of cardiovascular diseases, particularly ischaemic heart disease in adulthood [3]. Researchers emphasised the importance of maternal nutritional status. Both undernutrition and excessive body weight can lead to adverse outcomes, including metabolic disorders, impaired immune function, macrosomia, disturbances in carbohydrate metabolism, and intrauterine growth restriction (IUGR), and low birth weight. The concept eventually evolved into the Developmental Origins of Health and Disease (DOHaD) theory. The DOHaD paradigm highlights that early-life exposures have an impact on long-term health. Foetal programming can influence various aspects of development, including tissue function, metabolism, and structural changes in organs. These effects are mediated by epigenetic mechanisms, where exposure to specific environmental factors may contribute to changes in the epigenome. Consequently, these modifications alter gene expression without changing the underlying DNA sequence [4]. This narrative review aims to summarise current evidence on the relationship between maternal nutritional status and intrauterine programming, with particular attention to two major exposure categories: maternal undernutrition and maternal overweight or obesity. The review discusses selected epigenetic mechanisms, including DNA methylation, histone modifications and microRNA (miRNA) expression, and considers how these mechanisms may contribute to long-term metabolic, cardiovascular, neurodevelopmental and immune-related outcomes in offspring. A further aim is to highlight sex-specific responses to adverse intrauterine conditions and to identify clinical implications for preconception and prenatal prevention.

To identify relevant literature for this narrative review, comprehensive searches were conducted across the PubMed, Scopus, and Web of Science databases between January and March 2026. These databases were selected for their broad and complementary coverage of the biomedical and nutritional sciences literature. The search was restricted to English-language, peer-reviewed articles published between 2000 and 2026, with a particular emphasis on studies published after 2015. The primary search terms included combinations of maternal nutrition, undernutrition, obesity, gestational diabetes, intrauterine programming, DOHaD, epigenetics, DNA methylation, histone modifications, microRNA, and offspring health. Eligible study types included original research articles (observational, interventional, and experimental animal studies), systematic and narrative reviews, and meta-analyses. Articles were excluded if they were published before 2015 (except for seminal historical works) or focused solely on single-micronutrient supplementation.

Given the narrative design of this review, no formal quality assessment tool was applied; the strength and consistency of evidence are discussed throughout the text, with explicit distinction made between findings from human observational studies and those derived from experimental animal models.

## 2. Epigenetic Mechanisms Linking Maternal Nutrition with Intrauterine Programming

Epigenetics refers to mitotically, and in some cases potentially meiotically, heritable changes in gene regulation that occur without changes in the DNA sequence. These mechanisms allow environmental factors, such as maternal nutrition, to influence gene activity without altering the genetic code itself. During critical periods of pregnancy, when cells undergo rapid division, differentiation and organ-specific maturation, epigenetic regulation may be particularly sensitive to environmental signals [4].

One of the best-described epigenetic mechanisms is DNA methylation, which involves the addition of a methyl group to cytosine residues. During healthy development, DNA methylation is essential for regulating gene expression, including the inactivation of the X chromosome. Depending on the site of modification, DNA methylation may also lead to either the activation or inactivation of specific genes, which can subsequently contribute to the pathogenesis of diseases, such as cancer or metabolic disorders [5]. Maternal nutritional status has been associated with altered DNA methylation patterns. For instance, excessive intake of energy and fat leading to maternal obesity has been associated with altered DNA methylation in the promoter regions of genes regulating hypothalamic function. These epigenetic modifications may play a role in appetite dysregulation and have been linked to increased food intake and a higher risk of obesity in the offspring. However, evidence from human studies is still insufficient to establish a causal relationship [5,6].

Another important layer of epigenetic regulation includes post-translational histone modifications, such as acetylation, ubiquitination, and phosphorylation. These modifications influence chromatin accessibility and thereby regulate transcriptional activity. Histone acetylation, one of the best studied modifications, is associated with the opening of the chromatin structure, allowing for active transcription. These processes are regulated by enzymes such as histone acetyltransferases, deacetylases, methyltransferases, and demethylases. Nutritional factors play a significant role in regulating the activity of these enzymes [6,7]. Maternal diet also influences the availability of crucial cofactors and substrates required for histone modification, altering gene expression patterns in the foetus. These changes are linked to metabolic disorders, including insulin resistance, type 2 diabetes, and obesity. Furthermore, experimental studies conducted on animal models indicate that high-fat diets can lead to the dysregulation of histone-modifying enzymes [8,9].

In addition to DNA methylation and histone modifications, non-coding RNAs, particularly microRNAs, contribute to post-transcriptional regulation of gene expression. Altered miRNA expression has been reported in the context of maternal undernutrition, excessive gestational weight gain and maternal obesity, with potential effects on placental function, organ development and metabolic regulation in offspring [6,7]. The main epigenetic mechanisms involved in developmental programming and their potential associations with offspring health outcomes are summarised in Table 1.

Some epigenetic marks may persist beyond birth and contribute to long-term changes in tissue function. However, evidence for true transgenerational transmission in humans remains limited and should be distinguished from intergenerational exposure, in which the mother, foetus and foetal germ cells may be exposed at the same time. These concepts are related to the “thrifty phenotype” and “catch-up growth” hypotheses. The thrifty phenotype hypothesis proposes that nutrient deprivation during foetal life may induce adaptive metabolic changes that prioritise immediate survival. If this prenatal adaptation is followed by rapid postnatal weight gain or exposure to an obesogenic environment, the risk of insulin resistance, type 2 diabetes and related metabolic disorders may increase. Similarly, catch-up growth refers to accelerated postnatal weight gain, often observed in children born with IUGR or low birth weight. Although catch-up growth may support short-term growth recovery, excessive or rapid catch-up growth has been associated with higher cardiometabolic risk later in life [1,16]. Although this review mainly focuses on maternal nutritional status, paternal preconception nutrition may also influence offspring development through sperm-related epigenetic mechanisms, including DNA methylation and small non-coding RNAs [14]. However, the magnitude and clinical importance of these effects in humans remain uncertain.

It should be noted that findings from animal models may not fully translate to human physiology due to species-specific differences in placental structure, metabolic regulation, and developmental trajectories. In addition, findings from human observational studies need to be interpreted carefully, as postnatal factors such as nutrition, socioeconomic status, and lifestyle may also influence the observed associations.

## 3. Effects of Maternal Undernutrition

Maternal undernutrition before or during pregnancy is associated with adverse alterations in the intrauterine environment, primarily characterised by a limited and inadequate supply of essential nutrients. These restrictive exposures may profoundly impair foetal growth, alter organ development, disrupt metabolic regulation, and increase long-term disease susceptibility.

### 3.1. Growth Restriction and Glucose Metabolism

Maternal undernutrition during pregnancy is a significant determinant of intrauterine programming and is associated with a range of long-term health outcomes in offspring. One of the most prominent consequences of maternal undernutrition is intrauterine growth restriction (IUGR), characterised by impaired foetal growth and reduced birth weight [1,17]. Exposure to maternal undernutrition has been consistently linked to disturbances in glucose metabolism. These associations may reflect adaptive metabolic programming involving reduced insulin sensitivity and altered pancreatic function, including changes in β-cell mass, as suggested by both experimental and observational studies [15,18]. A reduced capacity for insulin secretion, combined with decreased insulin sensitivity, may contribute to the long-term dysregulation of glucose homeostasis.

### 3.2. Cardiovascular and Renal Consequences

Inadequate nutrient supply during critical stages of foetal development may also impair cardiac growth and vascular maturation, potentially contributing to persistent alterations in cardiovascular function observed in later life [19,20]. Both experimental and epidemiological evidence indicates that protein-energy deficiency during gestation disrupts cardiomyocyte development, leading to structural remodelling of the myocardium. Reported changes include alterations in sarcomere organisation and shifts in the expression of contractile proteins, particularly an increased ratio of β- to α-myosin. These modifications may impair cardiac contractility and reduce overall functional efficiency. In addition, increased expression of genes encoding collagen types I and III contributes to myocardial fibrosis through enhanced extracellular matrix deposition. This process is associated with an elevated number of fibroblasts and the progressive stiffening of cardiac tissue [19]. Vascular structure is also affected. Adverse intrauterine conditions are linked to increased intima–media thickness (IMT), a recognised marker of early vascular remodelling [20]. Elevated IMT reflects structural changes within the arterial wall and is highly predictive of an increased risk of atherosclerosis and cardiovascular events in later life. Renal development is similarly influenced by limited nutrient availability during foetal life. The disruption of nephrogenesis—a critical determinant of nephron endowment—has been linked to a reduced number of functional filtration units. A lower nephron count may increase functional demand on the remaining nephrons and promote compensatory hyperfiltration. Over time, this adaptive mechanism contributes to progressive renal damage and a gradual decline in kidney function. These structural adaptations are closely associated with an increased risk of hypertension [21]. Elevated intraglomerular pressure and altered renal haemodynamics may lead to long-term disturbances in blood pressure regulation. Furthermore, changes in gene expression within renal tissue indicate the involvement of epigenetic mechanisms in mediating these developmental effects [21,22]. Thus, early-life exposure to inadequate nutrient supply correlates with persistent cardiovascular and renal alterations, increasing susceptibility to hypertension, chronic kidney disease, and related complications in adulthood.

### 3.3. Hepatic Metabolism

Maternal undernutrition and IUGR may also affect hepatic development and long-term metabolic regulation. Experimental and clinical data suggest changes in the expression of genes involved in gluconeogenesis, lipid synthesis and insulin signalling [6,23]. These effects are partly mediated by epigenetic modifications, including altered DNA methylation patterns in key metabolic genes, such as the *Cyp7a1* gene. Consequently, offspring exposed to maternal undernutrition exhibit an increased risk of developing dyslipidaemia with elevated total cholesterol and low-density lipoprotein (LDL) levels. In addition, altered hepatic metabolism may contribute to systemic metabolic imbalance. Infants with low birth weight often exhibit increased activity of the somatotropic axis, including elevated insulin-like growth factor 1 (IGF-1). While this may temporarily support postnatal growth, the overarching adverse intrauterine environment induces persistent changes in liver function, potentially increasing the lifelong susceptibility to metabolic disorders [10,23].

### 3.4. Neurodevelopmental and Behavioural Outcomes

Beyond the metabolic and cardiovascular systems, inadequate nutrient supply during critical periods of foetal development may lead to long-term disturbances in brain development and function. The central nervous system is particularly sensitive to intrauterine conditions, as processes such as neuronal proliferation, differentiation, and maturation occur continuously during this period. Maternal undernutrition may impair these processes, potentially contributing to structural and functional alterations within the developing brain. These changes can affect regions responsible for cognitive function, memory, and behavioural regulation [24].

Exposure to IUGR has been associated with long-term impairments in cognitive performance. Affected individuals may exhibit deficits in learning ability, memory, and executive function, reflecting structural disturbances established during the initial stages of neurodevelopment. Behavioural issues have also been reported in individuals exposed to adverse intrauterine conditions. These include difficulties with attention, emotional regulation, and adaptive behaviour. The underlying mechanisms involve both the direct effects of inadequate nutrient supply and the indirect consequences of intrauterine stress. Alterations in the hypothalamic–pituitary–adrenal axis have been described, leading to long-term changes in the individual’s stress response. Dysregulation of this system may contribute to increased secretion of corticotropin-releasing factor (CRF) and adrenocorticotropic hormone (ACTH), with subsequent elevation of glucocorticoid levels. Prolonged exposure to glucocorticoids may contribute to further neuronal damage, particularly within the hippocampus. In addition, epigenetic modifications affecting neuronal gene expression solidify these persistent alterations. Overall, maternal undernutrition during pregnancy is associated with long-lasting neurodevelopmental and behavioural disturbances, potentially increasing the offspring’s vulnerability to cognitive deficits and behavioural disorders in later life [17,24]. It is important to note, however, that postnatal factors, including childhood nutrition, the socioeconomic conditions, and environmental stimuli, may either exacerbate or moderate these effects. Therefore, careful interpretation of observational data is necessary to disentangle these influences.

## 4. Effects of Maternal Overweight and Obesity

Maternal overweight and obesity before or during pregnancy are associated with alterations in the intrauterine environment, including increased availability of glucose, lipids, insulin-related signals and pro-inflammatory mediators. These exposures may influence foetal growth, body composition, metabolic regulation and long-term disease susceptibility.

### 4.1. Foetal Overgrowth and Glucose Metabolism

Gestational diabetes mellitus (GDM), which is closely associated with maternal overweight and obesity, represents an important and distinct mediator of adverse offspring outcomes. Offspring of mothers with GDM are at higher risk of developing obesity, metabolic syndrome and type 2 diabetes in later life [25,26].

One of the most characteristic outcomes of this environment is foetal overgrowth, clinically described as macrosomia. Increased glucose transfer through the placenta stimulates excessive foetal insulin secretion. During intrauterine development, this hormone acts as a potential growth factor, promoting cellular proliferation. Consequently, hyperinsulinemia is associated with increased adiposity and altered body composition at birth. Beyond its direct effects on physical growth, chronic exposure to elevated glucose concentrations may alter the development of metabolic regulatory pathways [27]. Macrosomia is also strongly associated with an increased risk of perinatal complications, including birth trauma and other delivery-related morbidities. Furthermore, alterations in pathways responsible for nutrient utilisation and storage can establish a long-term imbalance between energy intake and expenditure [27,28].

Chronic intrauterine exposure to elevated glucose concentrations may contribute to increased transplacental glucose transfer, potentially leading to foetal hyperglycaemia. These metabolic changes may persist beyond the intrauterine period and are associated with long-term disturbances in glucose homeostasis. Offspring exposed to excessive nutrient availability are therefore at a higher risk of developing insulin resistance, which represents a central mechanism in the pathogenesis of type 2 diabetes [11].

Evidence from human studies suggests that these long-term effects may be mediated, at least in part, by epigenetic modifications in the foetal epigenome. In a clinical study of 101 mother–infant pairs, GDM was associated with significantly lower DNA methylation of the MC4R gene promoter in newborns compared to those born to normoglycaemic mothers, while maternal obesity was associated with higher placental LPL methylation on the foetal side, correlating with elevated maternal LDL-cholesterol [29]. These findings suggest that GDM and maternal obesity may leave distinct epigenetic signatures with potential implications for the long-term metabolic health of the offspring; however, causal inference from observational data remains limited. Accordingly, these findings should be interpreted as evidence of association rather than proof of a causal mechanism.

### 4.2. Cardiovascular Consequences

Significant cardiovascular consequences have also been reported in the offspring of mothers with excessive body weight. Chronic exposure to elevated nutrient levels during foetal development can lead to early functional changes within the vascular system. Such conditions negatively impact blood vessel maturation, reducing the adaptive capacity of the arterial system and leading to long-term dysregulation of cardiovascular function. In addition, excessive maternal body weight can influence the mechanisms involved in blood pressure regulation. Altered intrauterine conditions may shift haemodynamic control, which is associated with increased vascular resistance and impaired regulation of arterial pressure. These adaptations strongly predispose individuals to the development of hypertension in later life, while structural changes within the vascular system may further contribute to an elevated cardiovascular risk associated with atherosclerosis [30].

### 4.3. Hepatic Metabolism and NAFLD

Increased availability of lipids and glucose during foetal development can also alter the metabolic processes of the liver, leading to long-term disturbances in hepatic function. Increased lipid transfer across the placenta may promote enhanced lipid accumulation within hepatocytes even during intrauterine life. This process may contribute to early alterations in hepatic lipid handling, creating imbalances between lipid uptake, storage, and utilisation. Consequently, the developing liver may exhibit a greater tendency for fat deposition. This predisposes the offspring to the development of non-alcoholic fatty liver disease (NAFLD), a condition characterised by excessive accumulation of lipids within hepatocytes. In the long term, such alterations may also contribute to dyslipidaemia and broader disturbances in metabolic regulation [31]. Impaired hepatic metabolism may exacerbate systemic metabolic imbalance, increasing susceptibility to insulin resistance and other components of the metabolic syndrome [31].

### 4.4. Inflammation, Immune Development and Microbiota

Furthermore, maternal obesity is frequently accompanied by a state of chronic inflammation during pregnancy. This inflammatory state affects the intrauterine environment and influences foetal development. Exposure to inflammatory mediators may interfere with the normal maturation of the immune system, leading to disturbances in immune regulation. This contributes to an enhanced inflammatory response and reduced ability to maintain immune homeostasis. Some immune-related changes may persist beyond the prenatal period and may contribute to later health outcomes [32]. In addition to immune alterations, changes in gut microbiota composition have been described in offspring exposed to excessive maternal body weight. The intrauterine environment and maternal metabolic status may influence early microbial colonisation, which plays a key role in the development of metabolic and immune functions. Differences in microbial profile include a higher abundance of bacteria from the phylum *Firmicutes* relative to *Bacteroidetes*. This maternal obesity-related dysbiosis may also affect the trajectory of the infant’s microbiome development [33,34]. However, attributing these microbial shifts solely to intrauterine programming is challenging, as the infant microbiome is profoundly shaped by postnatal variables, including the mode of delivery, breastfeeding practices, and early antibiotic exposure [35].

### 4.5. Neurodevelopment and Mental Health

Excessive maternal nutrient availability, inflammation and hormonal imbalance may also influence foetal brain development during sensitive developmental windows [36,37]. Offspring of mothers with overweight, obesity or diabetes have been reported to show a higher risk of neurodevelopmental and behavioural difficulties, including impairments in learning, memory, attention and emotional regulation [38,39]. Behavioural disturbances are equally prevalent, often manifesting as difficulties in maintaining attention, impaired emotional regulation, and altered behavioural responses. Such changes may be associated with disruptions in the neurobiological mechanisms responsible for adaptive behaviour and the body’s stress response. The underlying mechanisms are multifactorial and involve the combined effects of metabolic, hormonal, and inflammatory factors present throughout the pregnancy [38,40].

In addition to negative impacts on cognitive and behavioural functioning, maternal obesity is associated with an increased susceptibility to affective disorders, such as anxiety and depression in children. This is thought to be due to chronic inflammation, impaired leptin and insulin signalling, and dysfunction of the hypothalamic–pituitary–adrenal (HPA) axis. Preclinical studies have shown that offspring of obese mothers exhibit depressive tendencies and enhanced stress responses, which correlate with persistent changes in serotonergic and dopaminergic neurotransmission in limbic brain regions [39]. These findings expand the DOHaD framework beyond physical and physiological outcomes to include mental health consequences, which are now recognised as critical for overall wellness throughout life. However, the influence of postnatal environmental factors such as parenting style, childhood diet, physical activity, and socioeconomic status cannot be overlooked, and these variables should be carefully considered when interpreting the observed associations between maternal obesity and offspring neurodevelopmental outcomes. Recent reviews emphasise that these postnatal factors can either reduce or strongly mask the initial effects of intrauterine programming on mental health [36]. The major offspring outcomes associated with maternal undernutrition and maternal overweight or obesity are summarised in Table 2.

## 5. Sex-Specific Responses to Intrauterine Programming

An important aspect of intrauterine programming is its sex-specific nature. Male and female foetuses differ in growth trajectories, metabolic requirements, placental function and hormonal regulation, which may lead to different responses to the same intrauterine exposure. These differences are relevant for the interpretation of both experimental and clinical studies because pooling male and female offspring may mask biologically important effects [37,41]. The placenta is a central mediator of sex-dependent responses to the intrauterine environment. Some studies suggest that the placenta of males typically prioritises foetal growth and development to the detriment of the placental ability to adapt and change [42,43]. In contrast, female placentas are more plastic and better able to respond to challenging situations. As a result, male offspring may be more vulnerable to growth restriction, metabolic dysregulation or neurodevelopmental consequences after maternal undernutrition or metabolic disturbance, while female offspring may show different or partially compensatory adaptation patterns [12,37,41].

Epigenetic regulation of gene expression plays a pivotal role in these processes. Sex-specific patterns of DNA methylation, histone modifications, and microRNA expression have been observed in response to maternal nutritional status [12,44]. For instance, maternal undernutrition has been linked to DNA methylation changes in pathways involved in glucocorticoid signalling and metabolic regulation, with differences reported between male and female offspring [44]. Some studies suggest that these alterations may be more pronounced in male offspring, potentially contributing to their higher susceptibility to metabolic syndrome, cardiovascular disease, and neurodevelopmental disorders later in life [41,43]. On the other hand, female offspring may be relatively protected or may demonstrate alternative, compensatory adaptive mechanisms [37].

Additionally, sex hormones may further influence how the foetus develops and affect epigenetic programming. These hormonal factors interact with placental function and the maternal metabolic environment, creating a complex network of regulatory mechanisms that shape long-term health outcomes [41,44]. Therefore, future studies on intrauterine programming should report offspring outcomes separately by sex whenever possible. This approach may improve interpretation of heterogeneous findings and support the development of preventive strategies that consider biological sex as one determinant of disease risk rather than as a simple confounder.

## 6. Clinical Implications and Future Directions

The present narrative review emphasises the role of maternal nutritional status in shaping the long-term health outcomes of offspring through mechanisms of intrauterine programming. The findings summarised in this analysis indicate that both insufficient and excessive nutrient supply during pregnancy may contribute to persistent alterations affecting multiple organ systems. The observed effects appear to reflect the integrated adaptive responses of the developing foetus to adverse intrauterine conditions. These responses are considered adaptations that may optimise survival under specific, challenging intrauterine conditions. Epigenetic regulation has been proposed as one potential mechanism underlying the persistence of these effects across the lifespan. By modulating gene expression in response to environmental signals, epigenetic mechanisms may contribute to long-term physiological adaptation without altering the genetic code. Importantly, these modifications may not only stabilise the programmed phenotype but also influence how the individual responds to postnatal environmental exposure.

The interaction between prenatal programming and postnatal conditions represents a crucial element in the development of chronic disease risk. From a clinical perspective, these findings highlight the importance of early-life prevention strategies. Interventions targeting maternal nutrition should not be limited to preventing caloric or micronutrient deficiencies but should also address the growing prevalence of excessive body weight. Optimising maternal metabolic status, ideally beginning in the preconception period, may therefore represent a key opportunity to reduce the long-term effects of metabolic and cardiovascular diseases globally.

Although the evidence presented supports the DOHaD paradigm, there are a number of limitations in the methodology that should be considered. First, the majority of human studies have an observational nature, and no causal relationship can be established based on these studies. Second, it is challenging to distinguish the effects of intrauterine programming from those of the postnatal environment, as maternal nutritional status is often associated with other aspects of lifestyle and socioeconomic factors that also influence offspring health. For instance, Mendelian randomisation studies have demonstrated that the association between maternal body mass index and offspring adiposity is heavily confounded by shared genetics, suggesting that the direct causal intrauterine effect may be less pronounced than previously thought in observational cohorts [45]. Furthermore, while large-scale epigenome-wide association studies consistently identify altered DNA methylation signatures in cord blood associated with maternal exposures, evidence suggests that many of these epigenetic marks may not persist into later childhood, questioning their permanent mechanistic role in later-life disease [13]. Third, the translational significance of experimental animal models requires cautious interpretation. Future research should prioritise prospective cohort studies using detailed preconception and postnatal data, as well as randomised controlled trials of nutritional interventions, to establish causal pathways.

Advancing this field of research requires a more integrated approach that combines insights from developmental biology, clinical nutrition and everyday medical practice. Future investigations should focus on identifying specific, critical periods of susceptibility and further clarifying the exact mechanisms through which the intrauterine environment interacts with postnatal lifestyle factors. From a clinical perspective, current evidence supports several effective strategies, including screening of maternal nutritional status and body composition before conception, preferably during adolescence; personalised dietary counselling, considering both caloric intake and micronutrient adequacy (e.g., folate, vitamin B12, choline) and the quality of fats and carbohydrates; monitoring gestational weight gain based on pre-pregnancy body mass index (BMI); and incorporating paternal nutritional assessment into preconception care [2]. Integrating these components into routine prenatal care may help mitigate the adverse effects of programming, although further interventional studies are needed to establish causality and cost-effectiveness. Such efforts will be essential for the development of targeted interventions aimed at improving long-term health outcomes for future generations.

Overall, maternal undernutrition and maternal overweight or obesity represent opposite nutritional exposures, but both may alter foetal adaptation and long-term disease susceptibility. The associated phenotypes differ, with growth restriction and reduced nephron or metabolic reserve being more typical of undernutrition, and foetal overgrowth, adiposity and inflammatory-metabolic dysregulation being more typical of maternal obesity. In both settings, postnatal growth, nutrition and lifestyle can either amplify or attenuate prenatal susceptibility.

### Limitation of the DOHaD Literature

Several methodological limitations of the DOHaD literature warrant explicit acknowledgement. The majority of human studies are observational in design, and residual confounding remains a significant concern, as maternal nutritional status is closely intertwined with socioeconomic position, educational attainment and access to healthcare—factors that independently influence offspring health and are difficult to fully account for in epidemiological analyses. The shared family environment, encompassing postnatal diet, physical activity and psychosocial exposures, may further contribute to observed associations independently of intrauterine programming. These limitations underscore the need for cautious interpretation of associative findings and highlight the importance of well-designed interventional studies to establish causality.

## 7. Conclusions

Maternal nutritional status during pregnancy represents a key determinant of developmental programming, influencing long-term physiological regulation of the offspring. Both maternal undernutrition and excessive body weight may lead to persistent alterations in metabolic, cardiovascular, and neurodevelopmental processes. These effects may involve adaptive foetal responses and epigenetic mechanisms influencing gene expression, although direct causal evidence in humans is lacking. The interaction between prenatal programming and postnatal environment further modifies disease risk, highlighting the importance of early-life exposures. Consequently, optimising maternal nutrition both before conception and throughout pregnancy may constitute an essential strategy for improving long-term population health.

## Figures and Tables

**Table 1:** Epigenetic mechanisms potentially linking maternal nutritional status with offspring health outcomes.

	Mechanism		
Characteristic	DNA Methylation	Histone Modifications	microRNA Expression
**Maternal exposures**			
*Clinical metrics*	Pre-pregnancy BMI; gestational weight gain; maternal obesity	Maternal obesity	Maternal obesity; excessive gestational weight gain
*Dietary patterns*	High-fat diet	High-fat diet; protein-energy restriction	Protein-energy restriction
*Metabolic conditions*	Altered nutrient availability; maternal undernutrition	Experimental metabolic models	Altered nutrient availability
**Biological context**			
*Tissues/samples*	Placenta; cord blood	Experimental foetal tissues; metabolic organs	Maternal circulation; placenta; cord blood
*Representative targets*	Metabolic and developmental pathways	Chromatin regulation of metabolic genes	Post-transcriptional regulation of developmental and metabolic genes
**Outcomes and evidence**			
*Potential offspring outcomes*	Altered growth, adiposity, metabolic regulation	Insulin resistance, altered lipid metabolism, obesity risk	Altered placental function, organ development, metabolic regulation
*Evidence type*	Human observational and experimental studies	Mainly experimental studies	Human observational and experimental studies
*Overall strength of evidence*	Moderate	Limited	Moderate
*Main limitations*	Tissue specificity and limited causal inference	Limited direct evidence in humans	Heterogeneity of samples and methods
*References*	[5,6,10,11,12,13]	[11,12,14,15]	[6,7]

BMI: body mass index.

**Table 2:** Main offspring outcomes associated with maternal undernutrition and maternal overweight or obesity.

	Maternal Exposure	
	Maternal Undernutrition	Maternal Overweight or Obesity
**Dominant intrauterine condition**	Limited nutrient availability impaired placental nutrient transfer	Excess glucose, lipids and inflammatory mediators
**Main early outcome**	IUGR low birth weight	Foetal overgrowth macrosomia altered body composition
**Main long-term outcomes**	Insulin resistance, hypertension, renal vulnerability, dyslipidaemia, cognitive and behavioural difficulties	Obesity, type 2 diabetes risk, hypertension, NAFLD, immune dysregulation, neurodevelopmental and affective outcomes
**Key mediating pathways**	Adaptive metabolic programming, altered nephrogenesis, HPA-axis changes, epigenetic regulation	Foetal hyperinsulinaemia, inflammation, altered placental function, microbiota changes, epigenetic regulation
**Important modifiers**	Timing and severity of undernutrition, catch-up growth, postnatal diet, socioeconomic conditions	Maternal glycaemia, gestational weight gain, breastfeeding, postnatal diet, activity and adiposity
**References**	[1,3,4,8,10,15,16,17,18,19,20,21,22,23,24]	[1,2,3,5,6,7,9,11,25,26,27,28,29,30,31,32,33,34,35,36,38,39,40]

IUGR: intrauterine growth restriction; HPA: hypothalamic–pituitary–adrenal; NAFLD: non-alcoholic fatty liver disease.

## Data Availability

No new data were created or analysed in this study. Data sharing is not applicable to this article.

## Abbreviations

The following abbreviations are used in this manuscript:

ACTH	Adrenocorticotropic hormone
BMI	Body mass index
CRF	Corticotropin-releasing factor
DNA	Deoxyribonucleic acid
DOHaD	Developmental Origins of Health and Disease
HPA	Hypothalamic–pituitary–adrenal
IGF-1	Insulin-like growth factor 1
IMT	Intima-media thickness
IUGR	Intrauterine growth restriction
LDL	Low-density lipoprotein
MiRNA	MicroRNA
NAFLD	Non-alcoholic fatty liver disease
ncRNA	Non-coding RNA
